# MicroRNA 21 and microRNA 155 levels in resistant hypertension, and their relationships with aldosterone

**DOI:** 10.1080/0886022X.2021.1915800

**Published:** 2021-04-23

**Authors:** Sonat Pınar Kara, Gulsum Ozkan, Ahsen Yılmaz, Nergiz Bayrakçı, Savaş Güzel, Elif Geyik

**Affiliations:** a Department of Internal Medicine, School of Medicine, Tekirdağ Namık Kemal University, Tekirdağ, Turkey; b Department of Nephrology, School of Medicine, Tekirdağ Namık Kemal University, Tekirdağ, Turkey; c Department of Biochemistry, School of Medicine, Tekirdağ Namık Kemal University, Tekirdağ, Turkey; d Department of Biology and Genetics, Genometri Biotech, İzmir, Turkey

**Keywords:** Resistant hypertension, hypertension, miRNA 21, miRNA 155

## Abstract

**Aim:**

MicroRNAs (miRNAs) are non-coding RNA molecules that serve as regulators following gene expression transcription. While studies have investigated the role of miRNAs in the pathogenesis of essential hypertension (HT), very few have considered their place in the pathogenesis of resistant hypertension (RH). The purpose of this study was to investigate levels of miRNA 21 and miRNA 155 in RH and their relationships with aldosterone.

**Method:**

Thirty-two normotensive patients, 30 newly diagnosed HT patients, and 20 RH patients were included in the study. Patients’ demographic data were recorded, and office blood pressure measurement and 24-h ambulatory blood pressure monitoring (24-h ABPM) were performed. Blood specimens were collected for miRNA 21, miRNA 155 and aldosterone measurement. MiRNA 21 and miRNA 155 levels in the control and patient groups and their relations with other demographic and biochemical parameters were then subjected to analysis.

**Results:**

No difference was determined in miRNA 155 levels between the groups, but miRNA 21 and aldosterone levels were significantly higher in the RH group (*p* < 0.001 and <0.05, respectively). At correlation analysis, miRNA 21 exhibited positive correlation with aldosterone, age, office SBP, 24-h ABPM all-day SBP. A 9.6 copy/uL level for miRNA 21 predicted presence or absence of RH with 95% sensitivity and 71% specificity (AUC:0.823, 95% CI (0.72–0.92).

**Conclusion:**

The study results revealed significantly higher miRNA 21 and aldosterone in RH patients than in healthy individuals and newly diagnosed hypertensives.

## Introduction

Hypertension (HT) is an increasingly important public health problem in Turkey and worldwide. Undiagnosed HT patients or those who fail to meet target blood pressure (BP) values are exposed to significant morbidity and mortality. Despite technological advances in the diagnosis and treatment of HT, control rates are still quite poor, at approximately 35–50% in HT patients receiving treatment [1–3]. Resistant hypertension (RH) patients represent one part of uncontrolled HT patients. RH is reported at a range of 5–30%. Patients failing to meet target BP values despite use of at least three antihypertensives, one of which must be a diuretic, at maximum doses and in appropriate combinations, or use of at least four antihypertensives, are regarded as having RH. It is very important for pseudo-resistance HT to be excluded before true RH is diagnosed. The most common causes of pseudo-RH are inappropriate BP measurement in the office, lack of compliance with antihypertensive therapy, dietary noncompliance, inappropriate antihypertensive therapy combination, failure to use a sufficient dose, or the diuretic dosage being kept low. True RH rates once pseudo-resistance and white coat HT have been excluded is reported at <10% [1,4]. Patients with RH have an approximately 50% higher incidence of cardiovascular events than HT patients without RH [5]. The investigation of the pathogenesis and treatment of both essential HT and RH is therefore still ongoing.

The pathogenesis of RH is unclear, although the causes may include hypervolemia, neuronal sympathetic activation, undiagnosed secondary HT, and the use of various medications. Mechanisms implicated in the pathogenesis include increased aldosterone, sympathetic nervous system activation, and increased inflammatory mediators [1,4,6,7].

MicroRNAs are small, non-coding RNA molecules approximately 22 nucleotides in length. This molecular family performs a gene regulatory function after transcription [8]. Since their discovery more than 20 years ago, studies have shown that miRNAs are associated with several cardiovascular diseases, and particularly HT [8–10]. Current studies have also reported that miRNA 21 and miRNA 155 may be associated with the atherosclerotic process, neovascularization, and vascular remodeling [11–14]. In Romero et al.’s experimental study, miRNA 21 was shown to have a possible role in aldosterone synthesis in the adrenal glands [15]. Very few studies have examined the role of miRNAs in the pathogenesis of RH [16]. Our scan of the literature also revealed no previous studies investigating miRNA 21 and miRNA 155 levels in patients with RH. Since previous studies have shown that miRNA 21 and miRNA 155 levels increase in line with BP and are related to left ventricular hypertrophy, carotid intima media thickness, atherosclerosis, microalbuminuria, and vascular smooth muscle proliferation, we set out to investigate miRNA 21 and miRNA 155 levels in RH patients exposed to high BP.

The purpose of this study was to investigate the levels of miRNA 21 and miRNA 155 in RH, the mortality and morbidity rates of which are rising, and their relationship with aldosterone.

## Material and method

### Patient selection

Individuals aged over 18, who had been informed about the study and expressed verbal willingness to take part, and with sufficient intellectual capacity to provide a medical history and for performing 24-h ABPM, were enrolled. Pregnant women, and individuals with coronary artery disease, chronic liver disease, chronic obstructive pulmonary disease, pulmonary HT, secondary HT, or active infection were excluded. Office BP measurements and 24-h ABPM were performed on individuals presenting to the internal diseases and nephrology clinics for checkup purposes, meeting none of the exclusion criteria, and consenting to take part in the study. Thirty-two normotensive patients were enrolled as the control group. Thirty patients newly diagnosed with HT through office BP and 24-h ABPM were included in the newly diagnosed HT group. Twenty patients without BP regulation were included in the RH group (the details of the RH patients are set out below). Patients were asked to collect 24-h urine during 24-h ABPM. Sodium (Na), creatinine, and albumin excretion were studied from the 24-h urine specimens. Creatinine clearance, 24-h Na excretion and albuminuria were calculated from the 24-h urine specimens. Ethical approval for the study was granted by the Tekirdağ Namık Kemal University Medical Faculty ethical committee (no. 2017-06). The study commenced following receipt of informed written consent. Eighty-two patients were included in the study. Patients’ demographic parameters were recorded.

### Office blood pressure measurements

Office BP was measured following the European Society of Cardiology/European Society of

Hypertension (ESC/ESH) Guideline [1]. Briefly, BP was measured with the patient in a seated position after resting for at least 5 min, with the forearm unclothed and supported at the level of the heart, using a suitably sized cuff with the auscultatory method. BP was measured from both arms, a second measurement being performed after 1 min from the dominant arm once this had been identified. Patients with a mean value for the two measurements taken at a 1-min interval of ≥140/90 mmHg were regarded as hypertensive. Twenty-four hour ambulatory BP monitoring (24-h ABPM) was subsequently performed.

### 24-h Abpm

All 24-h ABPM measurements were taken with a Mobil-O-Graph NG 24 h ABPM Classic (I.E.M. GmbH, Stolberg, Germany) apparatus from the non-dominant arm in all cases. Patients were requested to note the numbers of hours spent sleeping, awake and eating, together with their daily activities. Daytime BP was measured at 15 min intervals and nighttime BP at 30-min intervals. Measurements exhibiting a minimum 70% validity from both day-time and nighttime measurements in 24-h ABPM records were subjected to analysis.

### Definition of resistant HT

Patients failing to meet target BP values despite using at least three antihypertensives, one a diuretic, at the maximum doses and in appropriate combinations, were regarded as RH [1]. Detailed histories were taken from all patients, including dietary habits (salt, alcohol, and licorice root consumption), drugs or substances used (narcotics, NSAIDS, oral contraceptives, etc.), sleep habits, and sleeping patterns. All resistant HT patients underwent pulmonary medicine examination in order to rule out Obstructive sleep apnea syndrome (OSAS). Exclusion was based on the OSAS Berlin questionnaire. Conditions causing pseudo-resistance were excluded. White coat HT was excluded in cases with a mean day-long 24-h ABPM value ≥130/80 mmHg following office measurements, and these were regarded as true RH. Creatinine, 24-h urinary albumin and Na excretion, fasting blood sugar, Na, potassium (K), calcium (Ca), complete blood count, and thyroid-stimulating hormone (TSH) tests were performed as recommended by the guidelines in order to screen for diseases capable of causing secondary HT [1]. Abdominal ultrasonography was performed on all patients. Patients with pseudo-resistant HT and secondary HT determined by screening tests were excluded from the study.

### Blood sample collection for biochemical tests

Blood specimens for biochemical tests were collected in the mornings after 8–12 fasting. Blood samples collected for aldosterone level measurement were collected after patients had remained standing for at least 30 min.

### Sample collection for miRNA measurement

Serum placed into gel-containing tubes was centrifuged and then transferred to new dry tubes and finally stored at −20 °C until laboratory examination. Serum samples were used for downstream processing.

### RNA extraction and measurement of RNA concentrations

RNA extraction was performed using a miRNeasy Serum/Plasma Kit (Qiagen, Germany) in line with the manufacturer’s instructions. RNA concentrations and purity were calculated using Quantifluor Handheld Fluorometers E6090 (Promega, USA) with a Quantifluor RNA System Kit as specified by the manufacturer.

### cDNA synthesis

The two miRNAs, miR-21 and miR-155, were quantified using ready to use Thermofisher Taqman miRNA assays (Thermo, USA), while specific RT primers contained in the assay were employed for reverse transcription with Bioline Sensifast cDNA Synthesis Kit Bioline, UK) to yield cDNA for both miRNAs. Briefly, 10 ng RNA is employed as a starting amount. The mix consists of 4 µL 5 × TransAmp buffer, 1 µL reverse transcriptase, 1 µL RT primers, 1 µL dNTP (10 mM each), and 10 ng RNA. The thermal cycling conditions in this study were 16 °C for 30 min, 42 °C for 30 min, 48 °C for 15 min, and 85 °C for 5 min).

### Absolute quantification of miRNAs

Absolute quantification of the two miRNAs, in the form of miRNA 21: hsa-miR-21, and miRNA 155 hsa-miR-155, was performed using a BioRad Droplet Digital PCR QX200 system (Bio-Rad, USA). Droplets containing the PCR mix and cDNA were generated. The PCR mix contained 12 µL ddPCR Supermix for Probes (No dUTP) (Bio-Rad, USA), 1 µL Taqman miRNA Assay (Thermo, USA), 10 µL RNAse, DNAse free water, and 2 µL cDNA. Generation of the droplets was carried out using 70 µL of Bio-Rad Droplet Generation Oil (Bio-Rad, USA) and 20 µL PCR mix with the help of special cartridges and a Bio-Rad Droplet Generator (Bio-Rad, USA). Subsequently, a total of 40 µL of the generated droplets was applied to PCR plates for PCR. Thermal cycling conditions of 95 °C for 5 min, 35 cycles of 95 °C for 30 s, 60 °C for 1 min, and 98 °C for 10 s were applied. The yielded PCR products were then placed in a QX200 Droplet Reader (Bio-Rad, USA). miRNA quantification was carried out using QuantaLife Software (Bio-Rad, USA). PCR products containing in excess of 10,000 droplets were subjected to analysis. If that number was not achieved, the experiment was repeated until sufficient droplets for absolute quantification could be obtained.

Quantification data were obtained with ddPCR, which permits the detection of droplets containing fluorescently labeled amplicons by means of laser. Calculation of these labeled droplets was conducted by Poisson Diagram *via* internal normalization of the findings.

**Figure 1.**
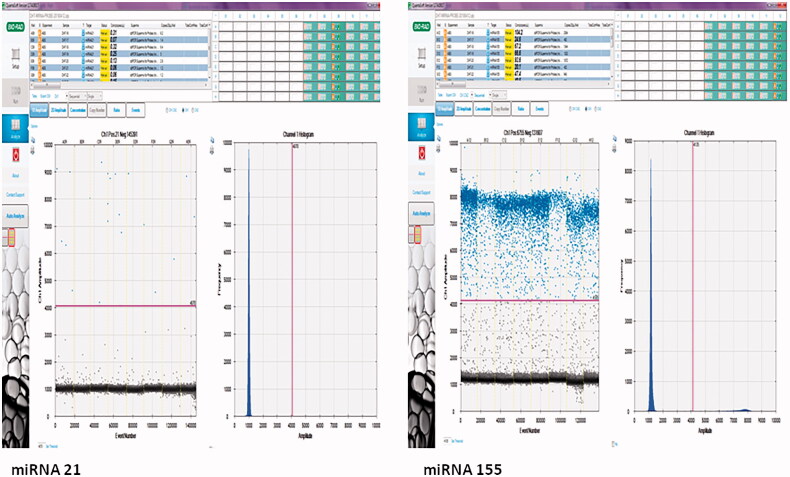
Droplet and copy numbers for miRNA 21 and miRNA 155. miRNA quantitation was performed under optimized ddPCR conditions using the Taqman assay. The results were presented as copy numbers per microliter. Samples with at least 10,000 droplets were included in the study. miRNA quantitation was calculated by separating negative (black) and positive (blue) droplets using a Poisson 95% confidence interval.

The uL in the copies/uL in miRNA abundance referred to the loaded nucleic acid in the ddPCR reaction, while in this study, it refers to the amount of cDNA product (Figure 1).

### ELISA

Peripheral blood specimens collected to obtain serum were centrifuged for 10 min at 2500× g. Following separation, serum specimens were stored at −80 °C until the day of study. Serum aldosterone levels were measured using a DiaMetra (DiaMetra S.r.l., Italy) ELISA kit based on the competitive ELISA principle (Catalog no. DCM053-11. Analytic sensitivity 14.6 pg/mL, intra-assay coefficient of variation (CV) < 10.4%, total CV < 13.9%).

### Statistical analysis

The Kolmogorov–Smirnov test was used to assess whether data were normally distributed. ANOVA was used to analyze normally distributed data at three-group comparisons, while the Kruskal Wallis test was used for non-normally distributed data. The chi square test was used for analysis of demographic characteristics, and Pearson correlation analysis was applied to determine correlations. Covariate analysis was applied in order to perform a comparison between the groups by removing the effect of BMI and age, associated with miRNA21 and emerging as significantly different between the groups at univariate analysis, on miRNA21 levels. Mean miRNA21 values were compared independently of the effect of BMI and age at covariance analysis. The sensitivity and specificity of miRNAs in diagnosing RH were assessed using receiver operator characteristics curve (ROC) analysis. *p* values <0.05 were considered statistically significant. Data were presented as mean ± SD or median (min–max)) All statistical analyses were performed on SPSS 20 software (IBM, Armonk, NY).

## Results

Thirty-two healthy controls (age 48.84 ± 9.35), 30 newly diagnosed HT patients (age 52.67 ± 7.49) and 20 RH patients (age 58.15 ± 11.05) were included in this study. Mean age and body mass index (BMI) were significantly lower in the control group than in the HT and RH patient groups (*p* < 0.006 and <0.05, respectively). Other demographic and biochemical parameters are shown in Table 1.

**Table 1.** A comparison of demographic and biochemical parameters in the hypertension, resistant hypertension and control groups.

	Control (*n* = 32)	Hypertension (*n* = 30)	Resistant HT (*n* = 20)	*p* Value
Age (year)	48.84 ± 9.35	52.67 ± 7.49	58.15 ± 11.05	<0.05
Gender (female %)	71.9	70	60	NS
BMI (kg/m^2^)	29.44 ± 4.92	30.69 ± 3.94	33.15 ± 4.89	<0.05
Office SBP (mmHg)	100 (90–120)	140 (130–160)	150 (140–170)	<0.001
Office DBP (mmHg)	62.5 (50–80)	90 (70–100)	90 (80–105)	<0.001
24-h ABPM SBP (mmHg)	109.56 ± 8.40	136.90 ± 11.13	143.15 ± 12.18	<0.001
24-h ABPM DBP (mmHg)	66.28 ± 5.71	83.20 ± 7.37	83.60 ± 9.89	<0.001
Creatinine (mg/dL)	0.73 ± 0.16	0.71 ± 0.13	0.82 ± 0.18	NS
Creatinine clearance (ml/min)	111.46 ± 18.15	113.50 ± 14.43	102.56 ± 11.81	NS
Glucose (mg/dL)	89.13 ± 7.58	107.40 ± 30.37	131.70 ± 39.91	<0.001
Sodium (mmol/L)	139.32 ± 1.70	139.56 ± 2.53	140.75 ± 3.22	NS
Potassium (mmol/L)	5.06 ± 0.41	5.01 ± 0.48	4.46 ± 0.35	<0.001
Calcium (mg/dL)	9.42 ± 0.31	9.57 ± 0.40	9.79 ± 0.31	<0.05
Uric acid (mg/dL)	4.67 ± 1.08	4.79 ± 1.17	6.08 ± 1.72	<0.05
Cholesterol (mg/dL)	203.1 ± 36.14	195.36 ± 40.85	223.5 ± 50.37	NS
Triglyceride (mg/dL)	180.50 (88–453)	180 (11–543)	196.50 (95.6–434)	NS
HDL-cholesterol(mg/dL)	41.6 ± 10.06	40.46 ± 11.30	44.15 ± 11.08	NS
LDL-cholesterol (mg/dL)	126 ± 31.10	114.16 ± 38.09	122.25 ± 35.20	NS
Hemoglobin(g/dL)	13.60 ± 1.51	13.83 ± 1.74	13.67 ± 2.47	NS
24h urine albumin (mg/day)	6.2 (0–57.5)	0 (0–156)	177(0–2940)	<0.001
24h urine sodium (mmol/day)	267.50 (45–775)	243.5 (69–440)	183 (107–405)	NS
MiRNA 21 (copy/uL)	2.6 (0–22)	3.2 (0–42)	18 (0–74)	<0.001
MiRNA 155 (copy/uL)	716 (0–2110)	651 (148–1680)	921 (0–2140)	NS
Aldosterone (ng/dL)	52.87 (5.23–427.69)	40.06 (1.01–108.44)	113.85 (13.80–556.5)	<0.05

Normally distributed data are expressed as mean ± SD, and non-normally distributed data as median (min-max) values.

**Figure 2.**
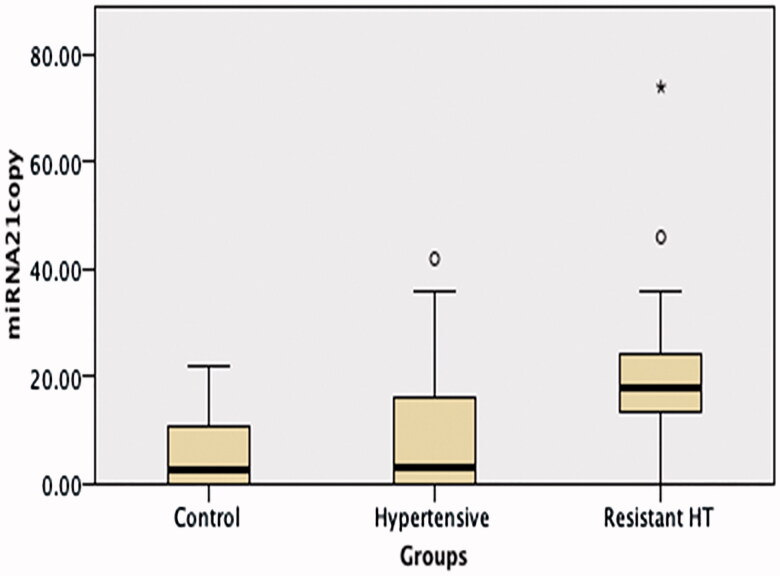
A comparison of miRNA 21 levels in the hypertension, resistant hypertension and control groups. miRNA 21 levels were significantly higher in the Resistant Hypertension group than in the other two groups (*p* < 0.001).

**Figure 3.**
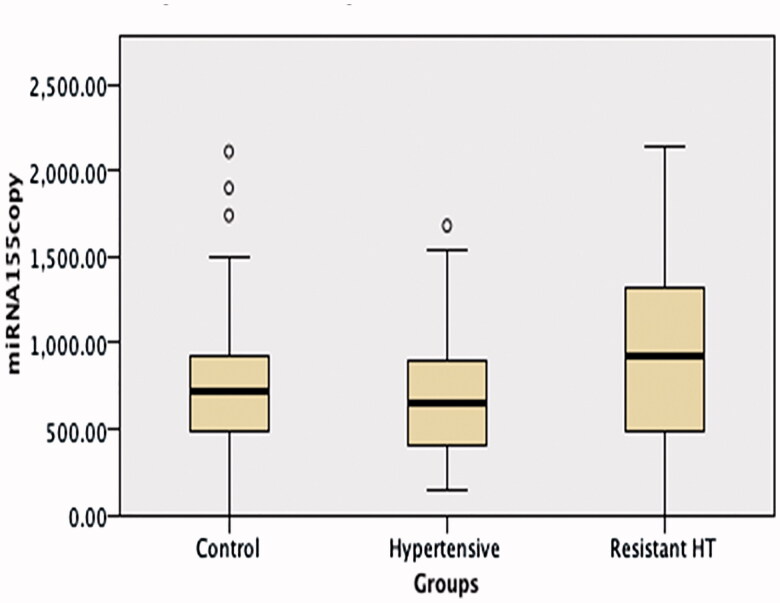
A comparison of miRNA 155 levels in the hypertension, resistant hypertension and control groups. No difference was observed between the resistant hypertension, hypertension and control group miRNA 155 levels.

No difference was observed between the RH, HT, and control groups’ miRNA 155 levels, but miRNA 21 and aldosterone levels were significantly higher in the RH group than in the other two groups (*p* < 0.001 and <0.05, respectively) (Table 1; Figures 2 and 3).

Patients in the RH group were using at least three antihypertensives, with 30% of patients using angiotensin converting enzyme (ACE) inhibitors, 70% angiotensin receptor blockers (ARBs), 100% thiazide group diuretics, 80% calcium channel blockers, 15% alpha blockers, 50% carvedilol, and 35% other beta blockers. No antihypertensives were being used when blood specimens were collected among newly diagnosed HT patients.

**Figure 4.**
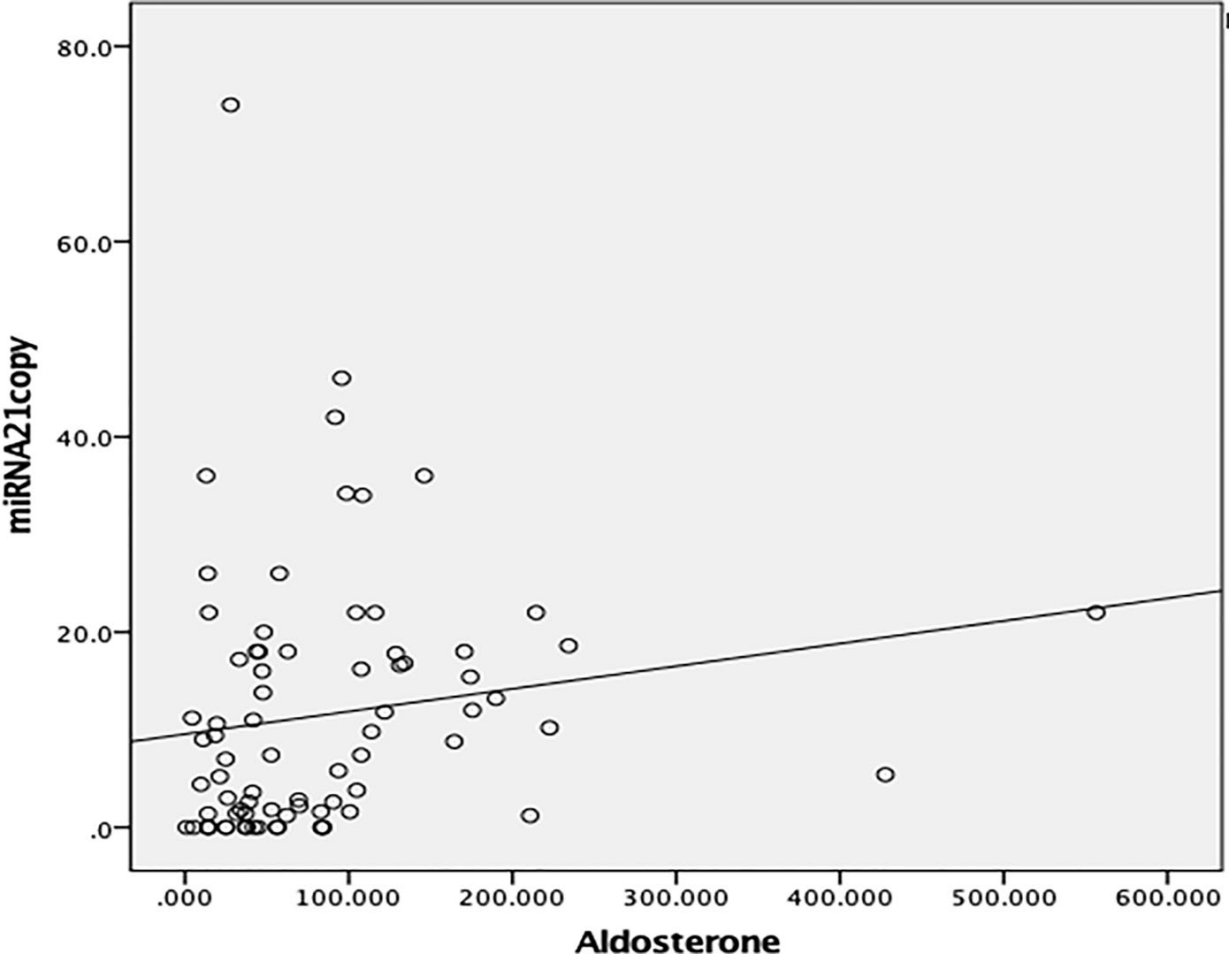
Correlation analysis of miRNA 21 and aldosterone. Positive correlation was determined between miRNA 21 and aldosterone in the patient group (*r* = 0.339, *p* = 0.002)

Analysis revealed that miRNA 21 exhibited positive correlation with aldosterone (*r* = 0.339, *p* < 0.05) (Figure 4), age (*r* = 0.237, *p* < 0.05), office SBP (*r* = 0.361, *p* < 0.05), 24-h ABPM all-day SBP (*r* = 0.306, *p* < 0.05), glucose (*r* = 0.323, *p* < 0.05), and urinary albumin (*r* = 0.319, *p* < 0.05), while miRNA 155 exhibited positive weak correlation only with aldosterone (*r* = 0.290, *p* < 0.05) and urinary albumin (*r* = 0.256, *p* < 0.05).

Mean miRNA 21 values were compared independently of the effect of age and BMI and covariance analysis. When the effects of age and BMI were excluded, we determined significant differences in miRNA 21 levels between the three groups (f: 8.42, *p* < 0.001 and f: 9.75, *p* < 0.001, respectively).

**Figure 5.**
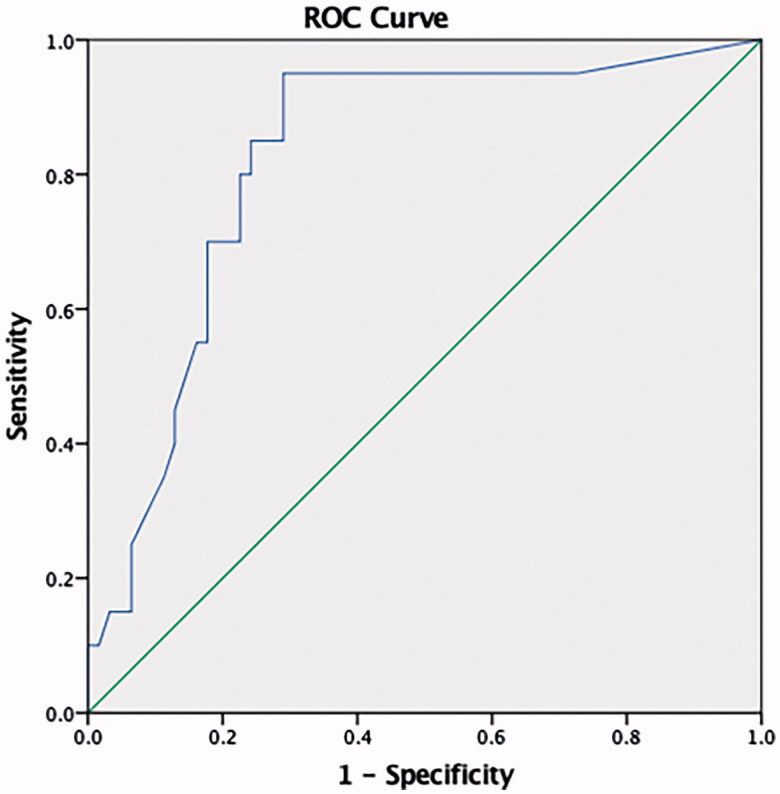
ROC curve analysis. MiRNA 21 was found to predict RH with 95 sensitivity and 71% specificity at a 9.6 copy/uL level (AUC: 0.823, 95% CI (0.72–0.92).

At ROC curve analysis, miRNA 21 was found to predict RH with 95 sensitivity and 71% specificity at a 9.6 copy/uL level (AUC: 0.823, 95% CI (0.72–0.92) (Figure 5). Aldosterone and miRNA 155 both exhibited low sensitivity and specificity.

## Discussion

The study results revealed significantly higher miRNA 21, aldosterone, age, 24-h urinary albumin, uric acid and BMI in RH patients than in healthy individuals and newly diagnosed hypertensives. miRNA 21 exhibited positive correlation with aldosterone, age, office SBP, 24-h ABPM SBP, glucose, and urinary albumin. At a 9.6 copy level, miRNA 21 predicted RH with 95% sensitivity and 71% specificity at ROC analysis. To the best of our knowledge, this is the first study to compare the relationships between RH and miRNA 21 and miRNA 155.

It is generally accepted that the prevalence of RH is not inconsiderable and that greater end-organ damage occurs in RH patients than in patients with controlled HT [7]. Unfortunately, the treatment of both RH and HT remains unsatisfactory in large numbers of patients, who require novel therapeutic agents. The pathogenesis of RH and the search for novel treatments based on that pathogenesis therefore remains a subject of great interest. Factors contributing to the development of RH include obesity, a high-sodium diet, excessive alcohol intake, the use of BP-raising medications and particularly non-steroidal anti-inflammatory drugs, inadequate diuretic therapy, and secondary HT. Leading causes of secondary HT include primary hyperaldosteronism, renal parenchymal diseases, renal artery stenosis, and OSAS [1,17,18]. The identification of aldosterone as an important factor inducing drug resistance represents a milestone in understanding the pathogenesis and treatment of RH. In previous studies, up to 20% primary hyperaldosteronism was detected in patients with RH [1,4,7]. In addition, the addition to medical treatment of an aldosterone receptor antagonist has been shown to produce marked BP control in RH patients, even if primary hyperaldosteronism is not present RH [19,20]. However, the contribution of aldosterone to the development of RH independently of plasma levels is controversial [7]. Consistent with the previous literature, aldosterone levels in the RH group in the present study were significantly higher than those in the control and newly diagnosed HT groups.

MicroRNAs are small, non-coding RNA molecules. One of the principal characteristics of the biology of miRNA is that a single miRNA is capable of targeting more than one gene, or that a single gene can be targeted by more than one miRNA [10]. miRNAs have therefore become the subject of research into complex pathophysiological processes in the pathogenesis of HT. Studies investigating the role of miRNAs in the pathogenesis of HT assume one of two forms. The first involves performing unbiased microarray scanning in the plasma of HT and healthy individuals, while the other involves scanning probable miRNAs in the plasmas of patients and healthy [21–24]. In our previous study of 52 controls and 51 HT patients under follow-up in the Cappadocia cohort, we investigated the place of miRNA 4516, miRNA 181a, miRNA 24, and miRNA 145 in the pathogenesis of essential HT, and showed that a decrease in miRNA 145 and an increase in miRNA 4516 may be involved [9]. In addition to examining the role of miRNAs in the pathogenesis of essential HT, studies have also investigated their relationship with end-organ damage [11,12,25]. Numerous studies have particularly evaluated the role of miRNA 21 in the pathogenesis of essential HT and its relationship with end-organ damage [26]. In their study of 28 HT and 28 healthy controls, Cengiz *et al.* determined high miRNA 21 levels in the HT patients and identified an association with carotid intima media thickness, a marker of subclinical atherosclerosis [11]. Yıldırım et al. similarly investigated 32 HT and 32 healthy controls and showed that miRNA 21 is associated with asymptomatic organ damage in the hypertensive population [12]. In their experimental study, Chen *et al.* determined an association between urinary miRNA 21 expulsion and renal histopathological damage in an induced HT rat model [27]. Experimental and clinical studies have also investigated the role of miRNA 155 in the pathogenesis of HT and its relationship with vascular smooth muscle proliferation [10,13,28]. Romano et al. [15] showed that miRNA-21 was up-regulated by the aldosterone secretagogue Ang II in H295R human adrenocortical cells, and that its up-regulation caused a specific increase in aldosterone secretion and cell proliferation. However, the number of studies investigating the levels and roles of miRNA-21 in the pathogenesis of RH is limited.

Sánchez-de-la-Torre et al. [29] assessed the relationship between plasma miRNA profiles and response to continuous positive airway pressure (CPAP) therapy in OSAS patients with RH. The identified three miRNAs, miRNA-378a-3p, miRNA-486-5p, and miRNA-100-5p, as determining response to CPAP therapy. In this study, miRNA 21 was significantly higher in the RH group than in the control and newly diagnosed HT groups. At a 9.6 copy level, miRNA 21 was capable of predicting presence or absence of RH with 95% sensitivity and 71% specificity at ROC analysis. The fact that miRNA 21 was also correlated with albumin showed that it may also be associated with end-organ damage. However, there was no difference in miRNA 155 levels between the control, newly diagnosed HT and RH groups in this study, and sensitivity and specificity were low at ROC curve analysis. We do not therefore think that miRNA 155 is associated with RH.

Although both were still within normal limits, Ca levels were higher, while K levels were slightly lower, in the RH group compared to the other groups in this study. We attributed this to greater use thiazide group diuretics in the RH group. Dose-dependent hypokalemia and hypercalcemia are frequently seen in patients using thiazide group diuretics [30]. Blood sugar was also higher in the RH group. This is due to the higher number of diabetic patients in the RH group. Numerous previous studies have shown a greater prevalence of HT and RH in diabetic patients [31].

This study has a number of strengths and weaknesses. One limitation is the relatively low patient number. Another is that the mean age of the RH group was higher than in the control and newly diagnosed HT groups. However, since RH increases with age, there is little possibility of matching the RH and control groups in terms of age. Most previous studies have also been unable to achieve this. Another limitation is that the majority of patients with RH were using multiple antihypertensives (ACE inhibitors, ARB, and thiazide diuretics) that affect renin levels, and since we were unable to discontinue, these we were unable to investigate renin levels and the aldosterone/renin ratio. The fact that we were ethically unable to discontinue these medications may also have affected our analyses. However, one particular strength of this study is that it is the first to investigate the levels of miRNA 21 and miRNA 155 in RH and their relationship with aldosterone.

In conclusion, HT and RH are an important public health problem. Many patients fail to be successfully treated. We think that when our findings are supported by further studies, a new horizon will open in terms of clarifying the role of miRNA 21 in the RH and perhaps in the development of new therapeutic targets.

## References

[1] Williams B, Mancia G, Spiering W, et al.; ESC Scientific Document Group. ESC/ESH Guidelines for the management of arterial hypertension. Eur Heart J. 2018;39:3021–3104. 30165516 10.1093/eurheartj/ehy339

[2] Chow CK, Teo KK, Rangarajan S, et al. PURE (Prospective Urban Rural Epidemiology) Study investigators. Prevalence, awareness, treatment, and control of hypertension in rural and urban communities in high-, middle-, and low-income countries. JAMA. 2013;310:959–968. 24002282 10.1001/jama.2013.184182

[3] Sengul S, Akpolat T, Erdem Y, Turkish Society of Hypertension and Renal Diseases, et al. Changes in hypertension prevalence, awareness, treatment, and control rates in Turkey from 2003 to 2012. J Hypertens. 2016;34:1208–1217. 26991534 10.1097/HJH.0000000000000901 PMC4856172

[4] Epstein M. Resistant hypertension: prevalence and evolving concepts. J Clin Hypertens (Greenwich). 2007;9(1 Suppl 1):2–6. 10.1111/j.1524-6175.2007.06171.x PMC8109975 17215648

[5] Daugherty SL, Powers JD, Magid DJ, et al. Incidence and prognosis of resistant hypertension in hypertensive patients. Circulation. 2012;125:1635–1642. 22379110 10.1161/CIRCULATIONAHA.111.068064 PMC3343635

[6] Wang Z, Peng X. Pathogenesis of essential hypertension: development of a 4-dimensional model. Hypothesis. 2013;11:1–7.

[7] Doroszko A, Janus A, Szahidewicz-Krupska E, et al. Resistant hypertension. Adv Clin Exp Med. 2016;25:173–183. 26935512 10.17219/acem/58998

[8] Romaine SP, Tomaszewski M, Condorelli G, et al. MicroRNAs in cardiovascular disease: an introduction for clinicians. Heart. 2015;101:921–928. 25814653 10.1136/heartjnl-2013-305402 PMC4484262

[9] Özkan G, Ulusoy Ş, Geyik E, et al. Down-regulation of miRNA 145 and up-regulation of miRNA 4516 may be associated with primary hypertension. J Clin Hypertens (Greenwich). 2019;21:1724–1731. 31556476 10.1111/jch.13704 PMC8030420

[10] Romaine SP, Charchar FJ, Samani NJ, et al. Circulating microRNAs and hypertension–from new insights into blood pressure regulation to biomarkers of cardiovascular risk. Curr Opin Pharmacol. 2016;27:1–7. 26827149 10.1016/j.coph.2015.12.002

[11] Cengiz M, Yavuzer S, Kılıçkıran Avcı B, et al. Circulating miR-21 and eNOS in subclinical atherosclerosis in patients with hypertension. Clin Exp Hypertens. 2015;37:643–649. 26114349 10.3109/10641963.2015.1036064

[12] Yildirim E, Ermis E, Allahverdiyev S, et al. Circulating miR-21 levels in hypertensive patients with asymptomatic organ damage. Medicine (Baltimore). 2019;98:e17297. 31574853 10.1097/MD.0000000000017297 PMC6775376

[13] Liu DF, Li SM, Zhu QX, et al. The involvement of miR-155 in blood pressure regulation in pregnant hypertension rat via targeting FOXO3a. Eur Rev Med Pharmacol Sci. 2018;22:6591–6598. 30402830 10.26355/eurrev_201810_16133

[14] Nosalski R, McGinnigle E, Siedlinski M, et al. Novel immune mechanisms in hypertension and cardiovascular risk. Curr Cardiovasc Risk Rep. 2017;11(4):12. 28360962 10.1007/s12170-017-0537-6 PMC5339316

[15] Romero DG, Plonczynski MW, Carvajal CA, et al. Microribonucleic acid-21 increases aldosterone secretion and proliferation in H295R human adrenocortical cells. Endocrinology. 2008;149:2477–2483. 18218696 10.1210/en.2007-1686 PMC2329274

[16] Parreira RC, Lacerda LHG, Vasconcellos R, et al. Decoding resistant hypertension signalling pathways. Clin Sci (Lond). 2017;131:2813–2834. 29184046 10.1042/CS20171398

[17] Myat A, Redwood SR, Qureshi AC, et al. Resistant hypertension. BMJ. 2012;345:e7473. 23169802 10.1136/bmj.e7473

[18] Aronow WS. Approaches for the management of resistant hypertension in 2020. Curr Hypertens Rep. 2020;22:3. 31916065 10.1007/s11906-019-1013-0

[19] Pimenta E, Gaddam KK, Pratt-Ubunama MN, et al. Aldosterone excess and resistance to 24-h blood pressure control. J Hypertens. 2007;25:2131–2137. 17885558 10.1097/HJH.0b013e3282a9be30

[20] de Souza F, Muxfeldt E, Fiszman R, et al. Efficacy of spironolactone therapy in patients with true resistant hypertension. Hypertension. 2010;55:147–152. 19858405 10.1161/HYPERTENSIONAHA.109.140988

[21] Li S, Zhu J, Zhang W, et al. Signature microRNA expression profile of essential hypertension and its novel link to human cytomegalovirus infection. Circulation. 2011;124:175–184. 21690488 10.1161/CIRCULATIONAHA.110.012237

[22] Yang Q, Jia C, Wang P, et al. MicroRNA-505 identified from patients with essential hypertension impairs endothelial cell migration and tube formation. Int J Cardiol. 2014;177:925–934. 25449503 10.1016/j.ijcard.2014.09.204

[23] Kontaraki JE, Marketou ME, Zacharis EA, et al. MicroRNA-9 and microRNA-126 expression levels in patients with essential hypertension: potential markers of target-organ damage. J Am Soc Hypertens. 2014;8:368–375. 24794206 10.1016/j.jash.2014.03.324

[24] Ceolotto G, Papparella I, Bortoluzzi A, et al. Interplay between miR-155, AT1R A1166C polymorphism, and AT1R expression in young untreated hypertensives. Am J Hypertens. 2011;24:241–246. 20966899 10.1038/ajh.2010.211

[25] Kontaraki JE, Marketou ME, Parthenakis FI, et al. Hypertrophic and antihypertrophic microRNA levels in peripheral blood mononuclear cells and their relationship to left ventricular hypertrophy in patients with essential hypertension. J Am Soc Hypertens. 2015; 9:802–810. 26358152 10.1016/j.jash.2015.07.013

[26] Li X, Wei Y, Wang Z. microRNA-21 and hypertension. Hypertens Res. 2018;41:649–661. 29973661 10.1038/s41440-018-0071-z

[27] Chen C, Lu C, Qian Y, et al. Urinary miR-21 as a potential biomarker of hypertensive kidney injury and fibrosis. Sci Rep. 2017;7:17737. 29255279 10.1038/s41598-017-18175-3 PMC5735153

[28] Xu D, Liao R, Wang XX, et al. Effects of miR-155 on hypertensive rats via regulating vascular mesangial hyperplasia. Eur Rev Med Pharmacol Sci. 2018;22:7431–7438. 30468491 10.26355/eurrev_201811_16283

[29] Sánchez-de-la-Torre M, Khalyfa A, Sánchez-de-la-Torre A, et al. Spanish Sleep Network. Precision medicine in patients with resistant hypertension and obstructive sleep apnea: blood pressure response to continuous positive airway pressure treatment. J Am Coll Cardiol. 2015;66:1023–1032. 26314530 10.1016/j.jacc.2015.06.1315

[30] Ellison DH, Loffing J. Thiazide effects and adverse effects: insights from molecular genetics. Hypertension. 2009;54:196–202. 19564550 10.1161/HYPERTENSIONAHA.109.129171 PMC2753383

[31] Lastra G, Syed S, Kurukulasuriya LR, et al. Type 2 diabetes mellitus and hypertension: an update. Endocrinol Metab Clin North Am. 2014;43:103–122. 24582094 10.1016/j.ecl.2013.09.005 PMC3942662

